# Habitual Use of Purgatives

**Published:** 1865-10

**Authors:** 


### Habitual use of Purgatives.

Dr. Radcliffe considers the habitual use of purgatives to be needless in all cases, and harmful in very many. He states that the bowels will act well enough if the diet be properly regulated; and he argues that the natural way of preventing constipation is to take care that the food be not deficient in oily and fatty matters, and (in suitable cases) in green meat. He states, also, that the cases in which purgatives are habitually resorted to are very generally cases of old age and of debility in various other forms—cases in which the constipation which it is intended to correct is a salutary condition rather than otherwise. He argues that the kind of diet most suited to the wants of the system in all these cases is one which (by the exclusion of the more indigestible and innutritious kinds of food) does not dispose to frequent stools, and in addition to this, that the same result is brought about in some measure by the digestion being much slower than it is in vigorous health. In these cases, in fact, he argues that constipation, within certain limits, is a state to be encouraged rather than a state to be corrected; and he protests against the common notion that the bowels ought to act every day, as a practice which is nearly as bad as that of Sancho Panza's mock doctor at Barataria, which was to take away the dish from the table before there had been time to make use of it.—*Med. Times and Gaz.*, August 19, 1865.—*Am. Medical Journal.*

